# Comparison of Long-Term Clinical Implications of Beta-Blockade in Patients With Obstructive Airway Diseases Exposed to Beta-Blockers With Different β1-Adrenoreceptor Selectivity: An Italian Population-Based Cohort Study

**DOI:** 10.3389/fphar.2018.01212

**Published:** 2018-10-25

**Authors:** Maurizio Sessa, Annamaria Mascolo, Cristina Scavone, Ilaria Perone, Annalisa Di Giorgio, Michele Tari, Annamaria Fucile, Antonella De Angelis, Daniel Bech Rasmussen, Magnus Thorsten Jensen, Kristian Kragholm, Francesco Rossi, Annalisa Capuano, Liberata Sportiello

**Affiliations:** ^1^Department of Drug Design and Pharmacology, University of Copenhagen, Copenhagen, Denmark; ^2^Department of Experimental Medicine, University of Campania “L. Vanvitelli”, Naples, Italy; ^3^Caserta Local Health Service, Caserta, Italy; ^4^Respiratory Research Unit Zealand, Department of Respiratory Medicine, Naestved Hospital, Naestved, Denmark; ^5^Department of Cardiology, Herlev and Gentofte University Hospital, Hellerup, Denmark; ^6^Department of Regional Health Research, University of Southern Denmark, Odense, Denmark; ^7^Department of Cardiology, North Denmark Regional Hospital, Hjørring, Denmark; ^8^Department of Cardiology, Aalborg University Hospital, Aalborg, Denmark

**Keywords:** clinical epidemiology, obstructive respiratory diseases, humans, pharmacoepidemiology, pharmacology, beta-blockers, heart failure

## Abstract

**Rationale:** Long-term clinical implications of beta-blockade in obstructive airway diseases remains controversial. We investigated if within the first 5 years of treatment patients with heart failure and obstructive airway diseases using non β1-adrenoreceptor selective beta-blockers have an increased risk of being hospitalized for all-causes, heart failure, and chronic obstructive pulmonary disease (COPD) when compared to patient using selective beta-blockers.

**Methods:** Carvedilol users were propensity matched 1:1 for co-treatments, age, gender, and year of inclusion in the cohort with metoprolol/bisoprolol/nebivolol users. Cox proportional hazard regression model was used to compare all causes, COPD, and heart failure hospitalization or the beta-blocker discontinuation between cohorts. For statistically significant associations, we computed the rate difference and the attributable risk.

**Results:** Overall, 11,844 patients out of the 51,214 (23.1%) were exposed to carvedilol and 39,370 (76.9%) to metoprolol/bisoprolol/nebivolol. Carvedilol users had a higher hazard for heart failure hospitalization (HR 1.29; 95% Confidence Interval [CI] 1.18–1.40) with 106 (95%CI 76–134; *p*-value < 0.001) additional cases of heart failure hospitalization per 10000 person-years if compared to metoprolol/bisoprolol/nebivolol users. In all, 26.8% (95%CI 22.5–30.9%; *p*-value < 0.001) of heart failure hospitalizations in the study population could be attributed to being exposed to carvedilol. Carvedilol users had a higher hazard (HR 1.06; 95%CI 1.02–1.10) of discontinuing the pharmacological treatment with 131 (95%CI 62–201; *p*-value < 0.001) additional cases of beta-blocker discontinuation per 10000 person-years metoprolol/bisoprolol/nebivolol users. In all, 6.5% (95%CI 3.9–9.0%; *p*-value < 0.001) of beta-blocker discontinuation could be attributed to being exposed to carvedilol.

**Conclusion:** On long-term follow-up period, carvedilol was associated with a higher risk of heart failure hospitalization and discontinuation if compared to metoprolol/bisoprolol/nebivolol users among patients with heart failure and obstructive airway diseases.

## Introduction

Beta-blockers are able to reduce mortality in patients with concurrent heart failure and respiratory diseases and, therefore, the co-existence of both diseases should not discourage the clinicians from using these drugs (Rutten et al., 2010; Short et al., 2011; Du et al., 2014; Lim et al., 2017). While beta-blockade is clinically useful for heart failure, it could induce bronchoconstriction and worsen lung function in patients with asthma or chronic obstructive pulmonary disease (COPD) (Grieco and Pierson, 2017). Among beta-blockers approved for heart failure, those less selective for β1-adrenoreceptor, such as carvedilol, were associated with a higher risk of obstructive airway diseases exacerbations (van der Woude et al., 2005; Hawkins et al., 2009; Short et al., 2011, 2014). In fact, European Society of Cardiology (ESC) and Global Initiative for Chronic Obstructive Lung Disease (GOLD) clinical guidelines recommended more selective β1-adrenoreceptor antagonist in patients with concurrent heart failure and respiratory diseases (i.e., COPD and asthma) (Dickstein et al., 2008, 2010; McMurray et al., 2012; Ponikowski et al., 2016). However, the majority of studies comparing β1-selective and non-selective beta-blockers in patients with heart failure and concurrent respiratory diseases focused on short-term follow-up period post beta-blockers initiation and only a few studies provided outcomes of public health interest (Salpeter et al., 2002; Hawkins et al., 2009; Jabbour et al., 2010; Rutten et al., 2010; Dungen et al., 2011; Lainscak et al., 2011; Etminan et al., 2012; Kubota et al., 2015; Ponikowski et al., 2016; Sessa et al., 2017a). Studies investigating clinical implications of beta-blockade on a long-term follow-up period are necessary, considering that the clinical implications observable during the start of beta-blocker initiation/up-titration do not necessarily reflect those occurring in the later phases of treatment (Baker and Wilcox, 2017). The first period of treatment with beta-blockers represents the most critical for bronchoconstriction because patients are up titrated to receive the optimal dosage of beta-blocker (Grieco and Pierson, 2017). In the later phases, when patients are symptomatically stable, there is a tendency to preserve a stable posological schema (Dickstein et al., 2008, 2010; McMurray et al., 2012; Ponikowski et al., 2016). Moreover, patients with asthma or COPD could have been exposed for a long period to β2-adrenoreceptor agonist, a phenomenon promoting the development of β2-adrenoreceptor desensitization in the respiratory system (Hausdorff et al., 1990; Lohse et al., 1996; Chong et al., 2003). Consequently, patients could be less sensitive to acute beta-blockade effects in the respiratory system due to both desensitization mechanisms of β2-adrenoreceptor and to the competitive binding of β2-adrenoreceptor agonist that are typically co-administrated in patients having both diseases. However, on long-term, the prolonged β2-adrenoreceptor antagonism performed by beta-blockers less selective for β1-adrenoreceptor receptors can alter pulmonary compliance leading to dynamic hyperinflation even in presence of β2-adrenoreceptor agonists (Jabbal and Lipworth, 2018). Dynamic hyperinflation, in turn, apart from negative respiratory outcomes, can promote impaired ventricular filling and low cardiac output along with a reduction of intrathoracic blood volume and pulmonary vein dimensions, all phenomenon that can promote decompensation of heart failure (Jorgensen et al., 2007; Watz et al., 2010; Barr et al., 2012; Stone et al., 2016; Watz, 2016). In this clinical scenario, β1-adrenoreceptor selectivity of beta-blockers may still play a key role for the recrudescence of both heart failure and obstructive airway diseases symptoms. To provide further insight on this aspect we investigated if patients with heart failure and concurrent COPD or asthma with long-term exposure to low β1-adrenoreceptor selectivity beta-blockers have an increased risk of being hospitalized for all-causes, heart failure, and obstructive airway diseases compared to non-selective beta-blockers.

## Materials and Methods

### Data Sources

Caserta Local Health Unit administrative databases were used as data sources. For reimbursement purposes, these databases contain information on age, gender, date of birth, and death, drugs redeemed/supplied and hospitalization occurred for more than 1,000,000 citizens living in the catchment area of Caserta (Campania Region, Italy). For each redeemed prescription, we retrieved information on the active ingredient, the Anatomical Therapeutic Chemical (ATC) classification code, the quantity, and the dispensing date. According to the Italian law for confidentiality data, in each administrative database, critical information for privacy are encrypted and a unique anonymized identifier guarantee the linkage among these databases.

### Study Population

The study population was composed of patients that redeemed carvedilol, metoprolol, bisoprolol or nebivolol prescriptions along with drugs for obstructive airway diseases (ATC R03) from December 31, 2010 to September 30, 2017. Given the different cardioselectivity of aforementioned β-blockers, which are all indicated for heart failure, patients were divided into two cohorts, those exposed to carvedilol (lower β1-adrenoreceptor selectivity) and those exposed to metoprolol/bisoprolol/nebivolol (higher β1-adrenoreceptor selectivity). We believe that by including patients co-exposed to reimbursed drugs for obstructive airway diseases (ATC R03) it is possible to identify both patients with asthma and COPD, however, mainly patients with COPD. In fact, Caserta Local Health Unit pharmaceutical administrative databases contain redeemed prescriptions for drugs that are reimbursed by the Italian Healthcare system. Those having ATC code R03 include aclidinium, beclomethasone, beclomethasone/formoterol, budesonide, budesonide/formoterol, fluticasone, formoterol, glycopyrronium, indacaterol, indacaterol/glycopyrronium, ipratropium bromide, nedocromil, salbutamol, salmeterol, salmeterol/fluticasone, theophylline, and tiotropium (Italian Medicine Agency, 2017). While some of these drugs are indicated for both asthma and COPD, the use of beta-blockers in asthma is contraindicated in both summaries of product characteristics and current clinical guidelines (Bousquet et al., 2007; Dickstein et al., 2008, 2010; McMurray et al., 2012; Ponikowski et al., 2016).

### Follow-Up Period

The first date of co-exposure to beta-blocker and R03 drugs was used as the index date for each patient. Patients were followed through Caserta Local Health Unit administrative databases from index date to outcome or censoring at death or at the end of follow-up on September 30, 2017.

### Outcomes

The first, second and third primary outcomes were the hazard ratio (HR) of being hospitalized for all causes, COPD and heart failure within 60 months from the index date for carvedilol users vs. metoprolol/bisoprolol/nebivolol users. The HR of discontinue the beta-blocker within 60 months from the index date for carvedilol users vs. metoprolol/bisoprolol/nebivolol users was also investigated.

### Study Covariates

Age, gender, and co-treatments were obtained at index date.

### Statistical Analyses

At index date, baseline characteristics of men and women were compared using the *t*-tests for continuous variables and χ^2^ test for categorical variables. To estimate the period of treatment with carvedilol or metoprolol/bisoprolol/nebivolol, the ongoing exposure was calculated for each individual by dividing the number of posological units dispensed by the estimated average dosage for the heart failure indication as described elsewhere (Andersson et al., 2012). Prior to data analyses, at index date, we matched patients exposed to carvedilol with those exposed to metoprolol/bisoprolol/nebivolol with a 1:1 match for our study covariates using the neighborhood matching algorithm (Austin, 2014). Histograms of propensity score were plotted prior and after matching to examine the effectiveness of the matching algorithm in balancing the covariates between the two cohorts. For the matched cohorts, cumulative incidence curves were generated to compare cumulative incidence for the study outcomes. Gray’s test was used to evaluate the hypotheses that cause-specific cumulative functions were equal. Cox proportional hazard regression model with only outcome and exposure was used to compare the hazard of all causes, COPD, and heart failure hospitalization or beta-blocker discontinuation between the two cohorts. For statistically significant associations, we computed the rate difference and the attributable risk percentage for the outcome under investigation for carvedilol users as opposed to metoprolol/bisoprolol/nebivolol users as described elsewhere (Davis et al., 2003; Xu et al., 2010). All analyses were based on intention-to-treat approach and used a statistically significant level of *p* < 0.05 (2-sided).

### Compliance With Ethical Standards

In Italy, retrospective register-based studies do not require informed consent or ethical approval (Agenzia Italiana del Farmaco, 2008).

## Results

In Caserta Local Health Unit (Italy) administrative databases, we identified 51,214 patients exposed to β-blockers for heart failure (carvedilol, metoprolol, bisoprolol and nebivolol) concurrently with obstructive airway diseases drugs (ATC R03). Out of the 51,214 patients, 11,844 (23.1%) were exposed to carvedilol and 39,370 (76.9%) to metoprolol/bisoprolol/nebivolol. By matching 1:1 carvedilol users with metoprolol/bisoprolol/nebivolol users, we obtained the study population of 23,688 patients (Table 1). The matching was successful and is presented in Table 1 and Supplementary Figure 1. Patients exposed to carvedilol were followed for 29,828 person-years, while those exposed to metoprolol/bisoprolol/nebivolol for 31,270 person-years in Caserta Local Health Unit administrative databases.

**Table 1:** Demographic characteristics and concurrent pharmacological treatments of patients exposed to carvedilol or metoprolol/bisoprolol/nebivolol in the period 2010–2017 among those assisted by general practitioners of Caserta Local Health Unit (Italy).

Variable	Level	Carvedilol (*n* = 11,844)	Metoprolol/bisoprolol/nebivolol (*n* = 11,844)	Total (*n* = 23,688)	*p*-value
Age	mean (SD)	74.4 (12.6)	74.2 (13.4)	74.3 (13.0)	0.188
Gender	Male	5858 (49.5)	5801 (49.0)	11659 (49.2)	0.466
Year of inclusion	2011	3131 (26.4)	2968 (25.1)	6099 (25.7)	
	2012	2916 (24.6)	2959 (25.0)	5875 (24.8)	
	2013	2067 (17.5)	2044 (17.3)	4111 (17.4)	
	2014	1259 (10.6)	1350 (11.4)	2609 (11.0)	
	2015	1146 (9.7)	1289 (10.9)	2435 (10.3)	
	2016	906 (7.6)	864 (7.3)	1770 (7.5)	
	2017	419 (3.5)	370 (3.1)	789 (3.3)	0.126
Angiotensin-converting enzyme inhibitors	N (%)	4211 (35.6)	1321 (36.5)	8532 (36.0)	0.264
Hydroxymethylglutaryl-CoA reductase inhibitors	N (%)	894 (7.5)	878 (7.4)	1772 (7.5)	0.711
Angiotensin II Receptor Blockers	N (%)	2726 (23.0)	2658 (22.4)	5384 (22.7)	0.139
Calcium channel blockers	N (%)	3075 (26.0)	3014 (25.4)	6089 (25.7)	0.372
Vitamin K antagonist	N (%)	941 (7.9)	958 (8.1)	1899 (8.0)	0.701
Low-dose acetylsalicylic acid	N (%)	4498 (38.0)	4460 (37.7)	8958 (37.8)	0.620
Loop diuretics	N (%)	4191 (35.4)	4268 (36.0)	8459 (35.7)	0.302
Potassium-sparing diuretics	N (%)	1827 (15.4)	1852 (15.6)	3679 (15.5)	0.666
Sodium-Channel Blockers (Class I Antiarrhythmics)^∗^	N (%)	252 (2.1)	260 (2.2)	512 (2.2)	0.754
Potassium-Channel Blockers (Class III Antiarrhythmics)^∗^	N (%)	580 (4.9)	595 (5.0)	1175 (5.0)	0.675
Organic nitrates	N (%)	1722 (14.5)	1785 (15.1)	3507 (14.8)	0.256
Anticholinergic long-acting	N (%)	2153 (18.2)	2126 (18.0)	4279 (18.1)	0.440
Anticholinergic short-acting	N (%)	566 (4.8)	582 (4.9)	1148 (4.8)	0.649
Beta 2 agonist + anticholinergic	N (%)				
Beta 2 agonist long-acting – inhalers	N (%)	584 (4.9)	611 (5.2)	1195 (5.0)	0.440
Beta 2 agonist short-acting – inhalers	N (%)	876 (7.4)	950 (8.0)	1826 (7.7)	0.075
Glucorticoid + beta 2 agonist	N (%)	7995 (67.5)	7950 (67.1)	15945 (67.3)	0.542
Glucorticoids - inhalers	N (%)	6715 (56.7)	6651 (56.2)	13366 (56.4)	0.409
Mast-cell stabilizer	N (%)	1745 (14.7)	1819 (15.4)	3564 (15.0)	0.184
Leukotriene receptor antagonist	N (%)	193 (1.6)	196 (1.7)	389 (1.6)	0.918
Phosphodiesterase inhibitors	N (%)	1557 (13.1)	1606 (13.6)	3163 (13.4)	0.359

*^∗^According to Vaughan-Williams classification of antiarrhythmic drugs (Vaughan Williams, 1984).*

### Comparison of All-Cause, Chronic Obstructive Pulmonary Disease, and Heart Failure Hospitalization Between Carvedilol and Metoprolol/Nebivolol/Bisoprolol Users

**FIGURE 1:**
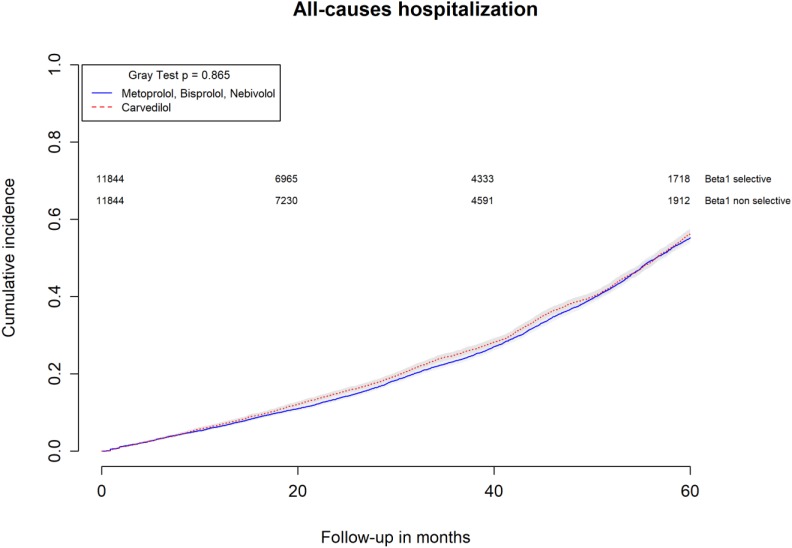
Cumulative incidence curves for all-cause hospitalization in carvedilol users vs. metoprolol, bisoprolol, and nebivolol users.

**FIGURE 2:**
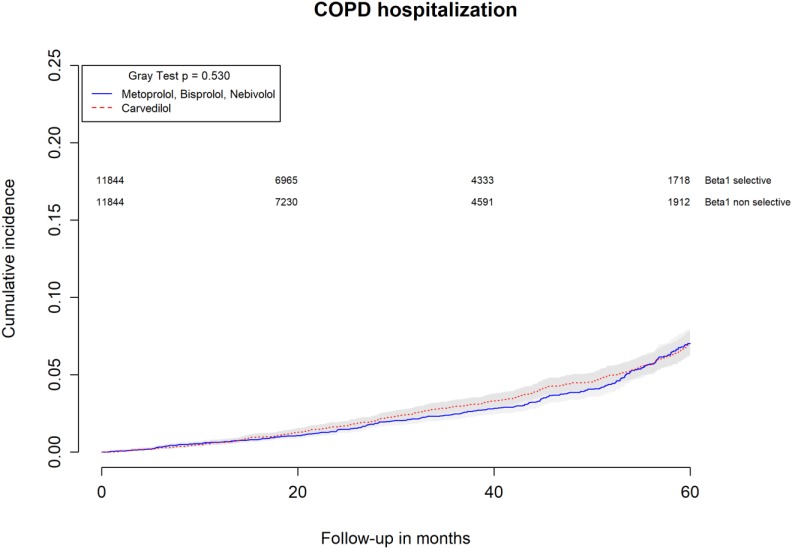
Cumulative incidence curves for COPD hospitalization in carvedilol users vs. metoprolol, bisoprolol, and nebivolol users.

**FIGURE 3:**
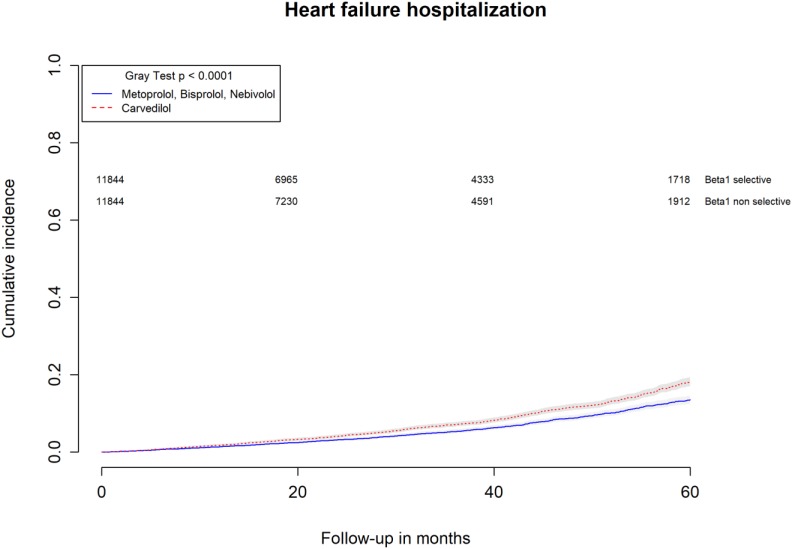
Cumulative incidence curves for heart failure hospitalization in carvedilol users vs. metoprolol, bisoprolol, and nebivolol users.

**FIGURE 4:**
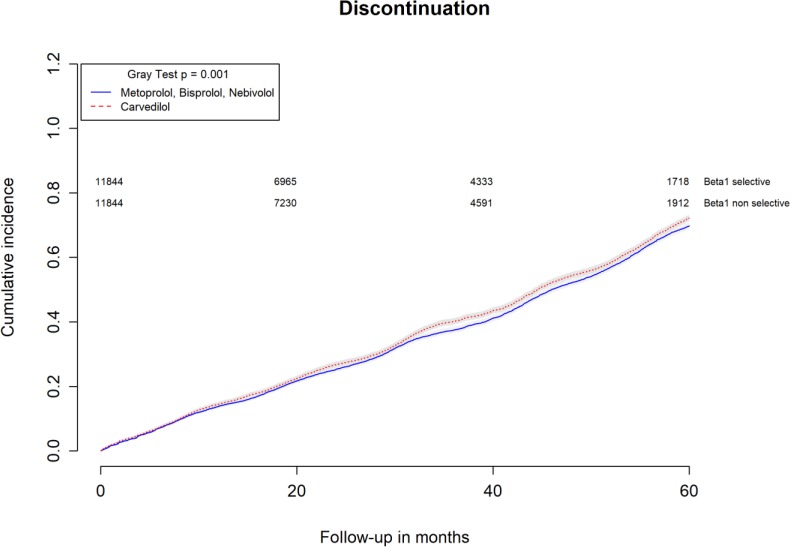
Cumulative incidence curves for beta-blocker discontinuation in carvedilol users vs. metoprolol, bisoprolol, and nebivolol users.

All-causes, COPD and heart failure cumulative incidence curves for carvedilol and metoprolol/bisoprolol/nebivolol users are provided in Figures 1–3, respectively. Within 60 months from the index date, no statistically significant differences were found for the hazard of being hospitalized for all causes (HR 1.00; 95% confidence interval (CI) 0.96–1.04) or COPD (HR 1.04; 95%CI 0.91–1.19) between carvedilol and metoprolol/bisoprolol/nebivolol users. For heart failure hospitalization, carvedilol users had a higher hazard compared with metoprolol, bisoprolol, and nebivolol users (HR 1.29; 95%CI 1.18–1.40). Additionally, within the same follow-up period, carvedilol users had a higher hazard (HR 1.06; 95%CI 1.02–1.10) of discontinuing the beta-blocker if compared to patients exposed to metoprolol/bisoprolol/nebivolol (Figure 4).

### Rate Difference and Attributable Risk Percentage of Heart Failure Hospitalization and Treatment Discontinuation for Carvedilol Users as Opposed to Metoprolol/Bisoprolol/Nebivolol

By computing the rate difference between the two cohorts, it was found that patients exposed to carvedilol had 106 (95%CI 76–134; *p*-value < 0.001) additional cases of heart failure hospitalization per 10,000 person-years when compared to patients exposed to metoprolol/bisoprolol/nebivolol. In all, 26.8% (95%CI 22.5–30.9%; *p*-value < 0.001) of heart failure hospitalizations were attributed to carvedilol exposure. Patients exposed to carvedilol had 131 (95%CI 62–201; *p*-value < 0.001) additional cases of beta-blocker discontinuation per 10,000 person-years when compared to patients exposed to metoprolol/bisoprolol/nebivolol. In all, 6.5% (95%CI 3.9–9.0%; *p*-value < 0.001) of beta-blocker discontinuation could be attributed to being exposed to carvedilol.

## Discussion

This study provided evidence on the impact of long-term beta-blocker administration for all causes, COPD, heart failure hospitalization or beta-blocker discontinuation within the first 60 months of treatment for carvedilol and metoprolol/bisoprolol/nebivolol users co-treated with drugs for COPD or asthma. On a long-term follow-up period, carvedilol use was associated with an increased hazard of heart failure hospitalization while no statistically significant differences were observed for all-cause and COPD hospitalizations. Additionally, carvedilol users were found to discontinue beta-blocker treatment more than metoprolol/bisoprolol/nebivolol users. Our results are in line with previous studies investigating this topic (Salpeter et al., 2002; Jabbour et al., 2010; Dungen et al., 2011; Lainscak et al., 2011; Etminan et al., 2012; Loth et al., 2014; Morales et al., 2014, 2017; Kubota et al., 2015; Baker and Wilcox, 2017; Sessa et al., 2017a) despite aforementioned studies evaluated different clinical outcomes and used different follow-up periods (Salpeter et al., 2002; Hawkins et al., 2009; Jabbour et al., 2010; Dungen et al., 2011; Lainscak et al., 2011; Etminan et al., 2012; Kubota et al., 2015; Ponikowski et al., 2016; Sessa et al., 2017a). Despite being unable to provide any causal explanation for our results, we believe that the clinical management of these patients may have played a key role in our findings. In fact, while in the start of the beta-blocker treatment patients tends to be highly monitored because of the higher risk of bronchoconstriction, in the later phases patients tend to be less strictly monitored (Gottlieb et al., 2002; Basile, 2003; Fonarow et al., 2008; Schimmer et al., 2009). Therefore, if in the later phases of beta-blocker treatment patients experienced worsening of the lung function it will not necessarily lead to an immediate modulation of the beta-blocker dosage which in turn potentially might happen at the next healthcare contact (Fonarow et al., 2008). Consequently, a sub-clinical effect of beta-blockade on the respiratory system summed with an ongoing exacerbation of the respiratory disease may results in a synergic negative effect in terms of respiratory symptomatology, which could result intolerable for patients and culminate in a hospitalization or beta-blocker discontinuation. A plausible explanation for why we observed an increased risk of heart failure hospitalization could be related to the prolonged β2-adrenoreceptor antagonism performed by beta-blockers less selective for β1-adrenoreceptor receptors. In particular carvedilol, as recently proved in clinical settings, can alter pulmonary compliance leading to dynamic hyperinflation even in presence of β2-adrenoreceptor agonists and corticosteroids (Jabbal and Lipworth, 2018). Dynamic hyperinflation, in turn, apart from negative respiratory outcomes, can promote impaired ventricular filling and low cardiac output along with a reduction of intrathoracic blood volume and pulmonary vein dimensions, all phenomenon that can promote decompensation of heart failure (O’Donnell et al., 2015) From clinical and public health perspectives, whenever confirmed with further evidence, this study opens new possibilities for improving the prognosis of beta-blocker treated patients with concurrent COPD or asthma, and to minimize the burden associated with their clinical management. We estimated that just by switching from carvedilol to metoprolol/bisoprolol/nebivolol it was theoretically possible to reduce 106 cases (95%CI 76–134) of heart failure hospitalization and 131 cases (95%CI 62–201) of beta-blocker discontinuation per 10,000 person-years of observational period. There is unfortunately only a speculative explanation of the results. The observation would benefit for additional data to investigate why increase hospitalization for heart failure, but not COPD is caused by the use of non-selective beta-blockers.

### Strengths and Limitations

Despite promising, our results should be considered in virtue of a set of limitations. The first is related to data sources. Caserta Local Health Unit administrative databases include information on drugs redemption and hospitalization occurred in in the catchment area of Caserta. Despite poorly likelihood, if a patient redeemed a prescription or if the person was hospitalized outside this area, the information is potentially lost. The second limitation is the limited access to historical data. We had less than a decade of observation period for our cohorts that do not permit the determination of the first hospitalization for a certain diagnosis (such as heart failure, asthma and COPD). In fact, we did not have the chance to assess for patients, the first date of beta-blocker administration and the date of their first hospitalization for heart failure or COPD. The third limitation is the lacking of indication of use for redeemed drugs that do not exclude the misclassification of some patients in our study population. In an example, it could not be ruled out that the indication of use of R03 drugs was for asthma instead of COPD. On the other hand, the major strengths of this study are the sample size, the long follow-up period, and inclusion of the entire population of Caserta minimizing the risk of selection biases. Moreover, the possibility of relying on real-world data from routine clinical practice which provide “evidence” that may directly support changes in clinical practice and policy decisions, especially in Caserta Local Health Unit (Romio et al., 2013).

## Conclusion

In the present study, patients with heart failure and concurrent respiratory disease were followed for 5 years. Carvedilol, a non-selective beta-adrenoreceptor blocker, was associated with an increased risk of discontinuation if compared to metoprolol, bisoprolol or nebivolol. Moreover, it was associated with an increased risk of heart failure hospitalization. We estimated that just by switching from carvedilol to metoprolol/bisoprolol/nebivolol it was theoretically possible to reduce heart failure hospitalization and beta-blocker discontinuation.

## Data Availability

The raw data supporting the conclusions of this manuscript will be made available from the authors on request, with legal reservations.

## Author Contributions

MS and AM developed the concept and designed the study. MS, AM, AC, and LS analyzed or interpreted the data. MS, AM, DR, MJ, AC, FR, and LS wrote the paper. All authors drafted the paper and revised it for important intellectual content and approved the final version of the manuscript to be published.

## Conflict of Interest Statement

The authors declare that the research was conducted in the absence of any commercial or financial relationships that could be construed as a potential conflict of interest.
