# Supplementary material for: Comparison of Long-Term Clinical Implications of Beta-Blockade in Patients With Obstructive Airway Diseases Exposed to Beta-Blockers With Different β1-Adrenoreceptor Selectivity: An Italian Population-Based Cohort Study
Source: Front Pharmacol. 2018 Oct 25;9:1212. doi: 10.3389/fphar.2018.01212 (PMC6232895; doi:10.3389/fphar.2018.01212)
Supplement: Supplementary file 1 [file Table_1.DOCX]

Supplementary Material

Comparison of long-term clinical implications of beta-blockade in patients with obstructive airway diseases exposed to beta-blockers with different 𝛽1-adrenoreceptor selectivity: an Italian population-based cohort study

Maurizio Sessa^1,2^⸸ & Annamaria Mascolo^2^⸸, Cristina Scavone^2^, Ilaria Perone^2^, Annalisa Di Giorgio^3^, Michele Tari^3^, Annamaria Fucile^3^, Antonella De Angelis^2^, Daniel Bech Rasmussen^4-6^, Magnus Thorsten Jensen^5^, Kristian Kragholm^7,8^, Francesco Rossi^2^, Annalisa Capuano^2^¥ & Liberata Sportiello^2^¥.

^1^Department of Drug Design and Pharmacology, University of Copenhagen, Copenhagen, Denmark
^2^Department of Experimental Medicine, University of Campania “L. Vanvitelli”, Naples, Italy.
^3^Caserta Local Health Service, Caserta, Italy.
^4^Respiratory Research Unit Zealand, Department of Respiratory Medicine, Naestved Hospital, Naestved, Denmark,
^5^Department of Cardiology, Herlev and Gentofte University Hospital, Hellerup, Denmark.
^6^Department of Regional Health Research, University of Southern Denmark, Odense, Denmark.
^7^Department of Cardiology, North Denmark Regional Hospital, Hjørring, Denmark
^8^Department of Cardiology, Aalborg University Hospital, Aalborg, Denmark

⸸ These authors contributed equally and served as co-first authors.

¥ These authors contributed equally and served as co-lead authors.

*** Correspondence:**Maurizio Sessa
maurizio.sessa@sund.ku.dk

**Keywords: clinical epidemiology_1_; obstructive respiratory disorders_2_; humans_3_; pharmacoepidemiology_4_; pharmacology_5_; beta-blockers_6_; heart failure_7_**


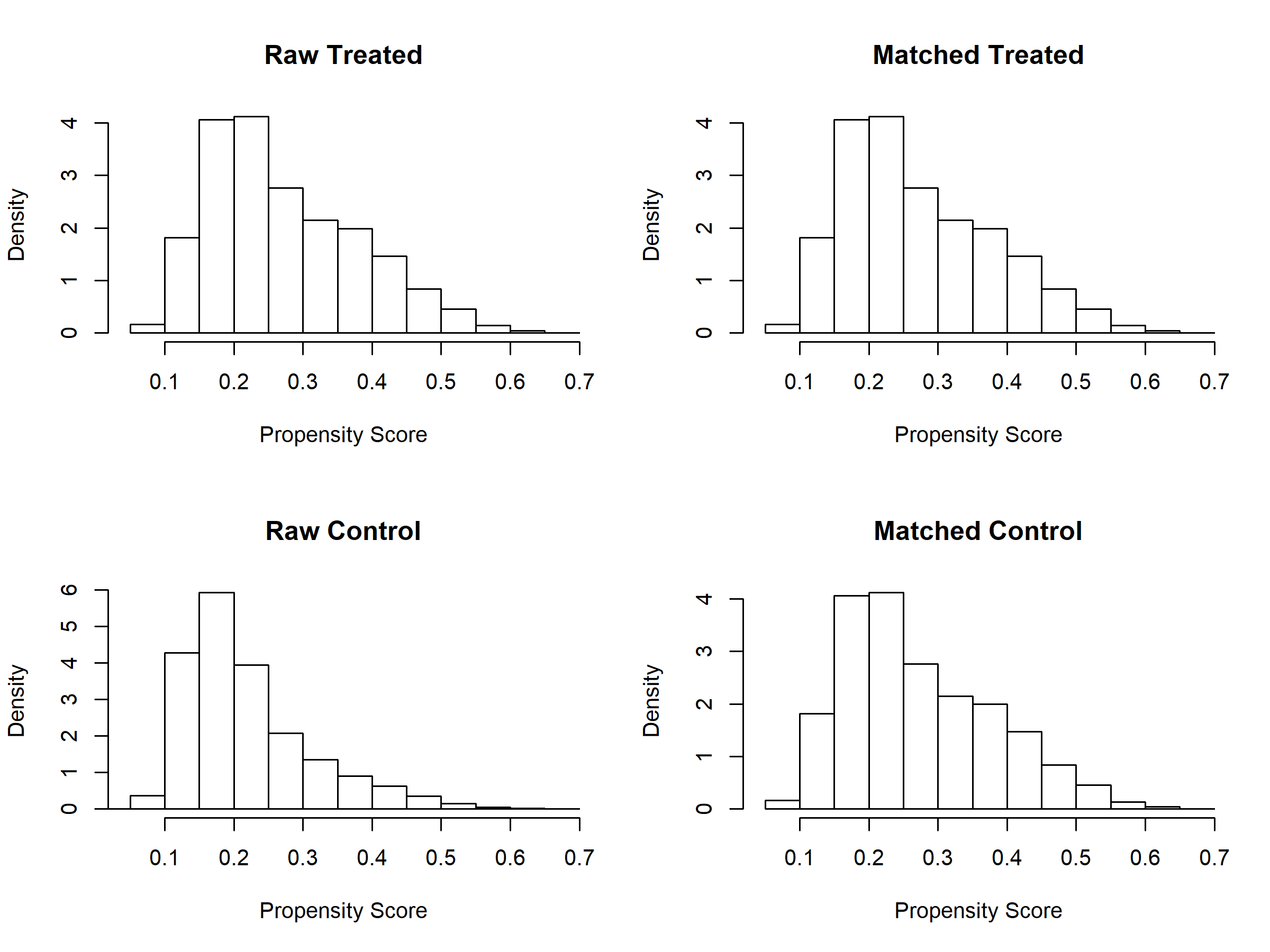


**Supplementary Figure 1**. Histograms of propensity score distance prior and after matching between the two cohorts. *Treated = patients exposed to carvedilol; Control = patients exposed to metoprolol/bisoprolol/nebivolol.*
